# Editorial: Toxicity of Pesticides on Health and Environment

**DOI:** 10.3389/fpubh.2018.00268

**Published:** 2018-09-19

**Authors:** Robin Mesnage, Gilles-Eric Séralini

**Affiliations:** ^1^Gene Expression and Therapy Group, Department of Medical and Molecular Genetics, Faculty of Life Sciences and Medicine, King's College London, London, United Kingdom; ^2^Committee for Independent Research and Information on Genetic Engineering (CRIIGEN), Paris, France; ^3^Network on Risks, Quality and Sustainable Environment MRSH, University of Caen Normandy, Caen, France

**Keywords:** toxicity, pesticides, multidi sciplinary work, regulatory toxicity, glyphosate

The aim of this research topic was to explore different aspects of the effects of pesticides on human health and the environment from a multidisciplinary point of view. The sustainability of agricultural cropping systems is a fundamental question on which the future of humanity is relying. Several indicators tend to suggest that the current system of agricultural production is reaching its limits and become unsustainable (Nicolopoulou-Stamati et al.). One hallmark of modern intensive agriculture, as well as a cause of farming system decline, is the intensive use of pesticides. They are used to kill insects, fungi or undesirable plants, reducing the biodiversity of agricultural landscapes to only one edible crop. This type of crop management has long-term detrimental effects on farming systems as the lack of biodiversity directly affects soil resilience.

Public policy is regularly shaken by health crises due to unexpected toxic effects of commonly used chemicals. This is the case for pesticides and their metabolites which can directly affect human and animal health (Nicolopoulou-Stamati et al.). Authors contributing to this research topic focused on pesticides associated to large scale cultivation of crops, for which the toxicity is debated, such as glyphosate-based herbicides (Cuhra et al.; Székács and Darvas) and neonicotinoids-based insecticides (Mullin et al.). It should also be borne in mind that the introduction of genetically modified (GM) crops at the end of the 1990s has considerably modified agricultural practices, including the use of pesticides. Almost all GM crops cultivated nowadays have been modified to tolerate an herbicide (mostly glyphosate-based herbicides) or/and produce their own modified insecticide. The toxicological properties of these insecticides is thoroughly addressed by Hilbeck and Otto in a review article, with a focus on combinatorial effects of Cry toxins.

The different studies published in our research topic shared a common conclusion. All revealed that the toxicity of pesticides is generally underestimated. For instance, pesticides are always commercialized as mixtures of different ingredients but only one declared of these ingredients is regulated and tested for human health effects. Ingredients such as surfactants, also named “inerts” or “formulants,” are poorly tested although they can be the most toxic ingredients in a pesticide formulation (1). This is clearly illustrated in the work by and colleagues, showing that organosilicone surfactants are potent standalone pesticides, and that they are toxic to honey bees (Mullin et al.). This work also shows for the first time that surfactant use could be linked with declining health of honey bee populations. Another important study investigated the inflammatory effects of a plant protection product, composed of crushed fenugreek seeds, on human peripheral blood mononuclear cells (Teyssier et al.). This work reminds us that although bio-based pesticides are of natural origin, direct toxicity of these products to human can be observed. They thus must be studied carefully to avoid non-target health effects as it is done for synthetic pesticides.

However, the problem goes beyond considerations on the toxicity of pesticides. It has social, political, ethical, and legal implications that could only be embraced through multidisciplinary research. Research on human health effects of environmental chemicals is highly specialized and few studies address the question from a multidisciplinary point of view. The debate on glyphosate is a topic for which multidisciplinary research bring meaningful insights. This idea is well supported by the analysis of the glyphosate case by Cuhra and colleagues arguing that specific aspects of the history, chemistry and safety of glyphosate and glyphosate-based herbicides should be thoroughly considered in present and future re-evaluations (Cuhra et al.). It is impossible to ignore structural changes in glyphosate uses. The use of glyphosate-based herbicides increased exponentially since their introduction on the market in the 1970s. It was amplified in the last decades by the introduction of agricultural genetically modified organisms (GMOs) designed to tolerate Roundup.

Perspectives from political economy are equally important. Glyphosate market is currently highly concentrated, and around 50% of global revenues are shared by only 4 companies. It has been estimated that Monsanto company made $4.76 billion in sales and $1.9 billion in gross profits from herbicide products, mostly consisting in Roundup (US securities and exchange commission, document 10-K, 1 mon-20150831x10k). It has been amplified now by the fusion with Bayer (2018). This may have critical consequences on political decisions related to the commercialisation of pesticides and GM crops designed to tolerate their residues. A similar line of thought is found in the perspective article published by Benbrook, describing 10 reforms and initiatives to create a more robust, science-driven regulatory infrastructure in the U.S.

Feeding 9 billion people or more with a healthy food through sustainable farming systems is one of the main challenges humanity has to face in the future. Agronomic and socioeconomic factors such as food availability, disparity in wealth, waste management, as well as dietary choices, are equally important to ensure global food security. A democratization of science is crucial in the current context of agricultural innovation that is increasingly driven by industrial interests (Vélot). Strategies to restore links between science, policy makers, and civil society are presented by (Vélot). This is well illustrated by the example of a participatory research project, in which the research work is shared between non-profit organizations from civil society or groups of citizens and academic researchers (from universities or major research organizations) like it was performed in CRIIGEN since 1999 (Vélot). In this line of though, the Cornell Alliance for Science launched an initiative in which “citizen scientists” are called upon to evaluate studies on health risks of GM crops and foods. The meaningfulness and limits of this project is examined by Antoniou and Robinson.

Our research topic confirms that new directions in agriculture are urgently needed to evaluate pesticide effects on health and environment. New agricultural policies should target sustainable development and protection of the consumers' health.

## Author contributions

All authors listed have made substantial, direct, and intellectual contribution to the work and approved it for publication.

### Conflict of interest statement

The authors declare that the research was conducted in the absence of any commercial or financial relationships that could be construed as a potential conflict of interest.
